# Downregulation of Spinal G Protein-Coupled Kinase 2 Abolished the Antiallodynic Effect of Electroacupuncture

**DOI:** 10.1155/2015/848603

**Published:** 2015-04-29

**Authors:** Huan Liu, Shen-Bin Liu, Qian Li, Huijing Wang, Yan-Qing Wang, Qi-Liang Mao-Ying

**Affiliations:** ^1^Department of Integrative Medicine and Neurobiology, State Key Laboratory of Medical Neurobiology, Institute of Acupuncture Research and Institutes of Brain Science, Collaborative Innovation Center for Brain Science, School of Basic Medical Science, Fudan University, Shanghai 200032, China; ^2^Department of Pharmacology, School of Basic Medical Science, Fudan University, Shanghai 200032, China

## Abstract

Acupuncture or electroacupuncture (EA) has been demonstrated to have a powerful antihypernociceptive effect on inflammatory pain. The attenuation of G protein-coupled receptor kinase 2 (GRK2) in spinal cord and peripheral nociceptor has been widely acknowledged to promote the transition from acute to chronic pain and to facilitate the nociceptive progress. This study was designed to investigate the possible role of spinal GRK2 in EA antiallodynic in a rat model with complete Freund's adjuvant (CFA) induced inflammatory pain. EA was applied to ST36 (“*Zusanli*”) and BL60 (“*Kunlun*”) one day after CFA injection. Single EA treatment at day 1 after CFA injection remarkably alleviated CFA induced mechanical allodynia two hours after EA. Repeated EA displayed significant antiallodynic effect from 2nd EA treatment and a persistent effect was observed during the rest of treatments. However, downregulation of spinal GRK2 by intrathecal exposure of GRK2 antisense 30 mins after EA treatment completely eliminated both the transient and persistent antiallodynic effect by EA treatment. These pieces of data demonstrated that the spinal GRK2 played an important role in EA antiallodynia on inflammatory pain.

## 1. Introduction

Patients diagnosed with trauma, inflammatory diseases, cancer, and diabetes often suffer from persistent pain. Chronic pain significantly reduces life quality of patients and brings a great challenge on clinical investigation. Acupuncture originated in ancient China has been proved to have a promising analgesic effect on several pain disorders, such as neuropathic pain, inflammatory pain, and cancer pain [1–3]. Electroacupuncture (EA), an important form of acupuncture, has been demonstrated to have an antiarthritic pain effect in monoarthritis rats by suppressing the proliferation of spinal microglia and decreasing the production of the proinflammatory cytokines including tumor necrosis factor- (TNF-) *α*, interleukin- (IL-) 1*β*, and IL-6 [4, 5]. EA treatment also inhibited the activation of microglial p38 mitogen-activated protein kinase (p38MAPK) and extracellular signal-regulated kinase (ERK) signaling and attenuated the neuropathic pain caused by spinal cord injury [6]. Consistently, evidence from last decades supported the anti-inflammatory and glial regulatory effect of EA [7–9]. However, the underlying mechanism has not been completely understood.

G protein-coupled receptor kinase 2 (GRK2) is a member of GRKs and is widely expressed in peripheral and central nervous system. GRK2 regulates cellular signaling by phosphorylating specific agonist-activated G protein-coupled receptors (GPCRs) [10, 11]. It was reported that the severity and extension of thermal hyperalgesia and mechanical allodynia induced by exogenous IL-1*β*, epinephrine, and prostaglandin E2 (PGE2) were enhanced in GRK2^+/−^ mice as compared to wild type mice [12–14], which suggested that GRK2 has a critical role in the molecular mechanisms subjacent in pathological pain processes. Additionally, the spinal GRK2 during inflammation was significantly decreased [14], while increased GRK2 expression significantly attenuated chronic pain [15]. Furthermore, downregulation of GRK2 increased the activation of spinal microglia and proinflammatory pathways and decreased the production of IL-10 from monocytes/macrophages [13, 14, 16]. GRK2 has also been demonstrated to directly interact with several downstream intercellular signaling pathways, including cAMP/Epac, p38 MAPK, phosphoinositide-3 kinase (PI3K)/Akt, ERK 1/2, and cytoskeletal elements [13–15]. But whether the spinal GRK2 contributes to the EA antiallodynic effect is still unknown.

The aim of present study was to investigate the possible role of spinal GRK2 in EA antiallodynic effect. For this purpose, low level of spinal GRK2 was induced by intrathecal administration with GRK2 oligonucleotides antisense, and the effect of low spinal GRK2 level on EA antiallodynic effect on inflammatory pain was evaluated in a rat model of complete Freund's adjuvant (CFA) induced mechanical allodynia. Mismatch sense was used as control.

## 2. Material and Methods

### 2.1. Animals

Experiments were performed on adult male Sprague-Dawley (SD) rats weighing 180–200 g. Animals were obtained from Shanghai Laboratory Animal Center, Chinese Academy Sciences, China. They were housed under a 12 : 12 hour light/dark cycle at a room temperature of 23 ± 0.5°C with food and water* ad libitum*. Prior to experimental manipulation, rats were habituated in the animal room for at least one week after delivery. All experiments were conducted in accordance with the National Institutes of Health Guide for the Care and Use of Laboratory Animals and the Ethical Issues of the International Association for the Study of Pain [17].

### 2.2. CFA Inflammatory Pain Model

CFA (Sigma, suspended in an 1 : 1 oil/saline emulsion, 0.1 ml, 50 *µ*g mycobacterium tuberculosis) was subcutaneously injected into the plantar surface of one hind paw of the rat to induce an inflammatory response. Normal saline in the same volume was set as control.

### 2.3. EA Treatment

**Figure 1:**
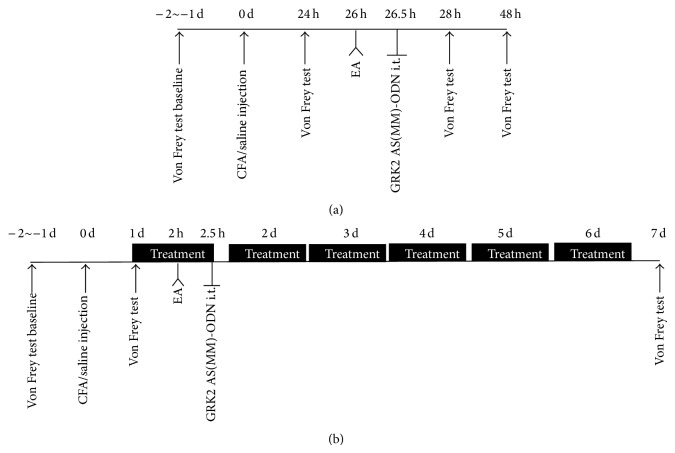
CFA injection, EA treatment, GRK2 AS(MM)-ODN i.t. injection, and behavioral test timeline for single (a) and repeated (b) treatment. The bold lines (b) were repetition of day 1.

EA treatment started at 24 hours after CFA injection. The body of rats was loosely immobilized while head and four limbs were kept free to move in a special designed holder. EA was administered by using two stainless steel acupuncture needles (0.3 mm in diameter) inserted into the bilateral “*Zusanli*” (ST36, 5 mm lateral to the anterior tubercle of tibia) at a depth of 7 mm and into “*Kunlun*” (BL60, at the ankle joint level and between the tip of the external malleolus and tendo calcaneus) at a depth of 5 mm. The two ipsilateral needles were connected to the output terminals of the HANS Acupuncture Point Nerve Stimulator (LH-202H Huawei Co., Ltd., Beijing, China). The EA parameters were as follows: square wave current output (pulse width: 0.2 ms), 1-2 mA (each intensity for 15 min); 2/100 Hz alternating frequencies (automatically shifting between 100 Hz and 2 Hz stimulation for 3 s each) [18]. The stimulation was given for 30 min, once per day for five consecutive days (Figure 1). Sham EA group animals received needle insertion subcutaneously into ST36 and BL60 in the same depth but without electrical stimulation.

### 2.4. GRK2 Antisense and Mismatch Oligodeoxynucleotides (ODNs): Preparation and Administration

In order to investigate whether the downregulation of spinal GRK2 would alter the antiallodynic effect of EA, GRK2 AS-ODN was intrathecally injected 30 min after EA treatment, and the mechanical threshold was measured. The GRK2 antisense oligodeoxynucleotide (AS-ODN) sequence 5′-CTTTTGGAAGATGTCG-3′, directed against nucleotides 480–495 of the rat GRK2 cDNA sequence, was synthesized by Sangong Biotech (Shanghai, CN). The mismatch (MM) ODN sequence was designed by mismatching seven bases (denoted by bold face) of the GRK2 AS sequence: 5′-GTTTACGTAGTTCTCC-3′ [19, 20]. Forty ng GRK2 AS-ODN was intrathecally injected 30 min after EA treatment. The same dose of MM ODN was delivered simultaneously as control.

### 2.5. Von Frey Test for Mechanical Allodynia

According to previous description [21], mechanical allodynia was measured using a series of von Frey hairs (0.4, 0.6, 1.4, 2.0, 4.0, 6.0, 8.0, and 15.0 g) (Stoelting, Wood Dale, Illinois, USA). Briefly, before the test, each rat was placed individually into a plexiglass chamber for 30 min acclimation. Then a von Frey hair was applied and held for approximately 5 to 6 seconds with a 10-minute interval between applications. A trial began with the application of 2.0 g von Frey hair. A positive response was defined as a brisk withdrawal of the hind paw upon stimulation. The testing contained five more stimuli after the first positive change in response occurred. Final score was converted to a 50% von Frey threshold using the Dixon up-and-down paradigm [22].

### 2.6. Western Blot Analysis

Western blot was conducted to verify the effect of AS-ODN on spinal GRK2 expression in normal rats. The L4-L5 segments of the spinal cord were quickly removed and ultrasonically disrupted in radioimmunoprecipitation assay (RIPA) lysis buffer (50 mM Tris (pH 7.4), 150 mM NaCl, 1% Triton X-100, 1% sodium deoxycholate, 0.1% sodium dodecyl sulfonate, sodium orthovanadate, sodium fluoride, ethylenediaminetetraacetic acid, and leupeptin), followed by centrifugation at 12,000 ×g. The total protein level in the supernatants was measured using the Pierce bicinchoninic acid (BCA) Protein Assay Kit (Thermo Scientific, Rockford, IL, USA). Samples were separated on 10% acrylamide gels and then transferred onto polyvinylidene fluoride membranes. After blocking with 5% skim milk in tris-buffered saline with Tween (TBST) (20 mM Tris–HCl, pH 7.5, 150 mM NaCl, and 0.05% Tween-20) for 2 h at room temperature, the membranes were incubated with the primary antibodies: rabbit anti-GRK2 (1 : 3,000, Santa Cruz) and mouse anti-GAPDH (1 : 10,000, Proteintech) at 4°C overnight. Then, the blots were washed in TBST and incubated in the appropriate secondary antibody (1 : 10,000, Abcam) for 2 h at room temperature. Western blot images were captured on an ImageQuant LAS4000 mini image analyzer (GE Healthcare, Buckinghamshire, UK), and the band levels were quantified using Quantity One version 4.62.

### 2.7. Statistical Analysis

All data are presented as mean ± standard error of the mean (SEM). The statistical significance of differences between groups was analyzed with Student's *t*-test or one-way analysis of variance (ANOVA) following the least significant difference (LSD) posttest or Bonferroni posttest. *P* < 0.05 was set as the threshold of significance.

## 3. Results

### 3.1. The Antiallodynic Effect of EA on Inflammatory Pain

**Figure 2:**
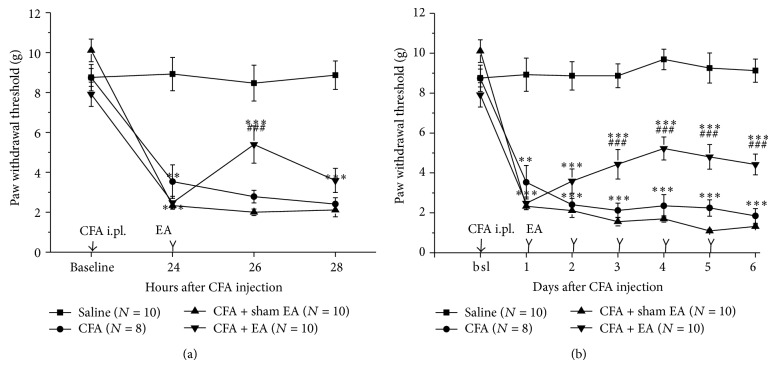
EA treatment attenuated the severity of mechanical allodynia inflammatory hypernociception induced by complete Freund's adjuvant (CFA). Single (a) and repeated (b) EA treatment statistically raised the decreased mechanical threshold. However, sham EA treatment did not exhibit any effect on paw withdrawal threshold (PWT). The data are expressed as the mean ± SEM (^∗^ *P* < 0.05, ^∗∗∗^ *P* < 0.001 versus saline group; ^#^ *P* < 0.05, ^###^ *P* < 0.001 versus sham EA group).

CFA i.pl. injection provoked a significant reduction in paw withdrawal threshold (PWT) one day after injection (Figure 2(a)), and PWT sustained in a low level in the following 5 days (Figure 2(b)). Single EA treatment 24 hours after CFA injection dramatically raised the PWT 2 h after treatment, but the antiallodynic effect was disappeared 24 hours after EA treatment (Figure 2(a)). Repeated EA treatments markedly reversed the reduced PWT by CFA from the second EA treatment and displayed a consistent antiallodynic effect during the following treatments (Figure 2(b)). However, no change on PWT was observed after sham EA treatment (Figure 2).

### 3.2. The Role of Spinal GRK2 in EA Anti-Allodynic Effect

**Figure 3:**
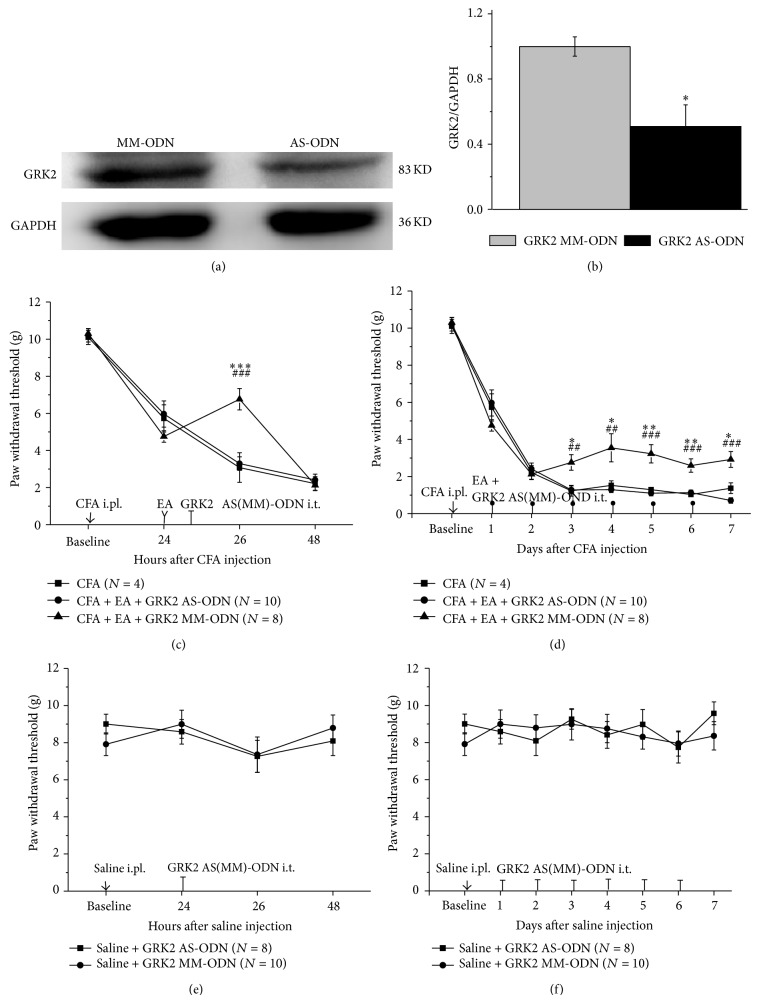
The attenuation of spinal GRK2 completely eliminated the antiallodynic effect of EA treatment. (a, b) Intrathecal injection of GRK2 AS-ODN significantly reduced the expression of GRK2, while GRK2 MM-ODN injection did not alter the production of GRK2. (c, d) Single and repeated exposure of GRK2 AS-ODN completely reversed the increase in paw withdrawal threshold (PWT) by EA treatment in CFA-induced mechanical allodynia. But GRK2 MM-ODN did not change the PWT after EA treatment. (e, f) Reduction of GRK2 by GRK2 AS-ODN did not alter the PWT in normal rats during single and repeated exposure to GRK2 antisense oligodeoxynucleotide. The data are expressed as the mean ± SEM (^∗^ *P* < 0.05, ^∗∗^ *P* < 0.01, and ^∗∗∗^ *P* < 0.001 versus CFA group; ^#^ *P* < 0.05, ^##^ *P* < 0.01, and ^###^ *P* < 0.001 versus CFA + GRK2 AS-ODN group).

Rats after i.t. treatment with GRK2 AS-ODN for three consecutive days showed a significant decrease in GRK2 protein levels in the spinal cord as compared to MM-ODN group (Figures 3(a) and 3(b)). Notably, the increase in PWT developed by EA treatment was completely reversed by i.t. injection of GRK2 AS-ODN as compared to MM-ODN treated rats or CFA rats without any treatment (Figures 3(c) and 3(d)). Single GRK2 AS-ODN i.t. significantly inhibited the transient effect of EA antiallodynic effect 2 h after AS-ODN injection (Figure 3(c)). And repeated GRK2 AS-ODN exposure suppressed the long-term antiallodynic effect of EA during all the experiments (Figure 3(d)). But GRK2 MM-ODN delivery displayed no effect on the mechanical threshold after EA treatment as compared to CFA rats. Furthermore, neither GRK2 AS-ODN nor MM-ODN changed the PWT on normal rats (Figures 3(e) and 3(f)). These pieces of data suggested that the attenuation of spinal GRK2 completely eliminated the antiallodynic effect of EA treatment. But, the expression level of GRK2 did not affect nociception in physiological conditions.

## 4. Discussion

Acupuncture has been demonstrated to exert a neuroprotective effect on several diseases especially for painful diseases. This is the first time to investigate the possible role of spinal GRK2 in acupuncture antiallodynic effect. In a rat model of CFA-induced inflammatory pain, single EA treatment displayed a transient antiallodynic effect 2 h after treatment, while repeated EA treatments significantly reduced the severity of mechanical allodynia in the following five days. The reduction of spinal GRK2 by GRK2 AS-ODN i.t. injection inhibited the transient effect and completely eliminated the consistent antiallodynic effect of EA.

Tissue injury or inflammation caused a robust release of proalgesic mediators which targeting selected GPCRs including C-C chemokine receptor 2 [23], neurokinin-1, lysophosphatidic acid [24], adrenergic, adenosine [25], and G protein-coupled EP-type receptors [26] and further led to the activation of nociceptive pathway [27]. These agonist-activated GPCRs could be moderated by GRK2. GRK2, also known as beta-adrenergic receptor kinase 1 (*β*ARK1), is the most studied member of a family of seven GRKs that are now known to regulate homologous desensitization of a wide array of GPCR [10, 11]. Previous work has shown that the level of GRK2 was significantly decreased in chronic neuropathic pain and inflammatory pain [20, 28]. Increased GRK2 level in the spinal cord significantly alleviated the pain behavior [15]. On the other hand, *μ*-opioid receptor (MOR) could be partly regulated by GRK2 through phosphorylation, which led to rapid endocytosis and desensitization. However, this process was followed by slow resensitization and recycle within an hour when exposed to endogenous opioids [29]. Moreover, the disruption of endogenous GRK2 function did not affect the endocytosis of endogenous MOR [30], which may be associated with GRK3- and/or GRK5-dependent desensitization of MOR [31]. In this study, the rats displayed significant mechanical allodynia after i.pl. CFA injection but significant increase in mechanical thresholds after EA treatment. However, the anti-EA effect was tested at 2 or 24 hrs after i.t. GRK2 AS-ODN, and the data show i.t. that administration with GRK2 AS-ODN after EA treatment significantly blocked the EA antiallodynic effect on inflammatory pain. Thus, the results indicated that the spinal GRK2 played an important role in the EA antiallodynic effect on inflammatory pain and the increase of GRK2 may not affect the inhibitory nociceptive pathway mediated by endogenous opioids.

As a component of the innate immune system, microglia samples the extracellular space of central nervous system through continuous extension, retraction, and remodeling of the cellular processes. Microglia responded quickly after injury or inflammation and took strong responsibility in neuroinflammation. Resting microglia undergo rapid morphological and functional activations [32, 33]. Active microglia exerted a cytotoxic effect due to the secretion of ROS and the proinflammatory cytokines including TNF-*α* and IL-1*β* and was responsible for painful behavior induced by inflammation and injury [34, 35]. It has been reported that the GRK2 in spinal microglia may play an important role in regulating pain procedure and is believed to be a key regulator in the transition from acute to chronic pain [36]. Low GRK2 level in microglia/macrophages significantly prolonged the pain behavior after acute administration with carrageenan, IL-1*β*, and CCL3 [14, 37]. However, another study showed that the downregulation of sensory neuronal GRK2 is responsible for constant pain procedure induced by PGE2 and epinephrine [12, 13]. Furthermore, intracisternal infusion of microglia inhibitor minocycline prevented the mechanical allodynia and rescued the reduction of neuronal GRK2 caused by alveolar nerve injury, indicating the glia-neuron cross-communication in GRK2-mediated nociceptive transmission [38].

Accumulated data demonstrated that EA attenuated pain through the inhibition of neuroinflammation by regulating spinal microglia. Previous studies have shown that nerve inflammation and nerve injury caused rapid activation of spinal microglia and elevated expression of TNF-*α*, IL-1*β*, and IL-6 [32, 33, 39], which promoted the nociceptive transmission in the spinal cord. EA treatment could inhibit the activation of spinal microglia and the occurrence of inflammatory events by blocking nuclear factor-*κ*B, ERK, p38 signaling, and the expression of downstream proinflammatory cytokines, including TNF-*α*, IL-1*β*, IL-6, and PGE2 [5, 9, 40, 41]. Thus, EA treatment significantly relieved the mechanical allodynia and thermal hyperalgesia induced by inflammation and nerve injury [2, 4, 5, 8]. The antihypernociceptive effect can be mimicked by intrathecal exposure with microglia inhibitor minocycline or glial inhibitor fluorocitrate [4, 5]. The present study is the first evidence of GRK2 in EA antihypernociception. But whether microglial or neuronal GRK2 contributes to EA antihypernociception on inflammatory pain needs further investigation.

## 5. Conclusion

The attenuation of spinal GRK2 completely reversed both the transient and long-term antihypernociceptive effect by EA treatment on inflammatory pain. The results further supported that spinal GRK2 may be a key molecular target for inflammatory pain regulation and developing strategies targeting GRK2 may be a promising way for clinical pain interventions.
